# Complete Genome of Bacillus megaterium Podophage Pookie

**DOI:** 10.1128/genomeA.01432-14

**Published:** 2015-01-29

**Authors:** Tsonyake N. Ladzekpo, Andrew J. DeCrescenzo, Adriana C. Hernandez, Gabriel F. Kuty Everett

**Affiliations:** Center for Phage Technology, Texas A&M University, College Station, Texas, USA

## Abstract

Bacteriophage Pookie is a novel podophage, isolated from soil, which infects Bacillus megaterium. B. megaterium is an important host for large-scale recombinant protein production. Here, we present the complete genome of phage Pookie and describe its core features.

## GENOME ANNOUNCEMENT

Bacillus megaterium is a Gram-positive bacteria widely used in industry due to its efficient protein secretion system and ability to grow on various inexpensive carbon sources (1). Additionally, it is nonpathogenic and lacks endotoxins (2). Bacteriophages infecting B. megaterium are readily found in the environment and may be able to provide biotechnological advances. With that in mind, we describe here the genome of novel B. megaterium podophage Pookie.

Bacteriophage Pookie was isolated from a soil sample collected in College Station, TX. Phage DNA was sequenced in an Illumina MiSeq 250-bp paired-end run with a 550-bp insert library at the Genomic Sequencing and Analysis Facility at the University of Texas (Austin, TX). Quality-controlled trimmed reads were assembled to a single contig at 67.3 fold coverage using Velvet version 1.2.10. The contig was confirmed to be complete by PCR using primers that face the upstream and downstream ends of the phage DNA. Products from the PCR amplification of the junctions of concatemeric molecules were sequenced by Sanger sequencing (Eton Bioscience, San Diego, CA). Genes were predicted using GeneMarkS (3) and corrected using software tools available on the Center for Phage Technology (CPT) Galaxy instance (https://cpt.tamu.edu/galaxy-public/). Morphology was determined using transmission electron microscopy performed at the Texas A&M University Microscopy and Imaging Center.

Pookie has a 40,214-bp genome with a G+C content of 40.57%. It has a coding density of 96.6% and encodes 52 predicted coding sequences. Pookie shares 89.6% nucleotide sequence identity to B. megaterium podophage Pony (NC_022770) according to an Emboss stretcher analysis (3). Pookie has a limited host range and infects B. megaterium Km Sp-.

Genes encoding proteins necessary for phage replication, morphology, gene regulation, and lysis were identified. Replication and recombination proteins include ssDNA-annealing (RecT-like) and binding proteins, a DnaC-like replication protein, a DnaB/DnaD-like replication protein (IPR006343) (4), and a plasmid replication/relaxation protein. Pookie also encodes two proteins containing lambda CI and Cro-like helix-turn-helix DNA-binding domains (IPR001387). The plasmid replication/relaxation protein and repressor-like DNA-binding proteins may suggest that Pookie may be a temperate phage that exists as a plasmid-borne lysogen. Few morphogenesis proteins were identified and include a capsid protein, tailspike, and tail fiber. The capsid protein was predicted using the HHpred server for protein homology detection and structure prediction (5). To accomplish DNA packaging, Pookie encodes large and small terminase subunits and a head-to-tail joining (portal) protein. Large terminase homology suggests that Pookie packages its DNA in a *pac* headful mechanism. As a *pac*-type phage, the circularly permuted genome of Pookie was opened to the small terminase gene by precedent (6). The genes which affect lysis encode a holin, anti-holin, and an endopeptidase endolysin. Pookie also codes for a SpoIIIE-like protein. SpoIIIE is responsible for packing DNA into the forespore in sporulating cells, but its role in phage infection has not been studied (7).

### Nucleotide sequence accession number.

The genome sequence of phage Pookie was contributed to GenBank under the accession no. KM236248.
